# Comparative Efficacy of PD‐1 Inhibitor‐Based Neoadjuvant Chemoimmunotherapy Regimens for Resectable Stage II–IIIa NSCLC: A Real‐World Retrospective Study

**DOI:** 10.1111/1759-7714.70123

**Published:** 2025-07-07

**Authors:** Bo Yan, Xiaoxuan Sun, Yan Sheng, Ran Zhang, Yanjun Su, Yulong Chen

**Affiliations:** ^1^ Department of Radiotherapy National Clinical Research Center of Cancer, Tianjin Medical University Cancer Institute and Hospital Tianjin China; ^2^ Department of Thoracic Cancer Tianjin Cancer Hospital Airport Hospital National Clinical Research Center for Cancer Tianjin China; ^3^ Department of Pathology Tianjin Cancer Hospital Airport Hospital National Clinical Research Center for Cancer Tianjin China; ^4^ Department of Lung Cancer Tianjin Medical University Cancer Institute & Hospital, National Clinical Research Center for Cancer, Tianjin's Clinical Research Center for Cancer, Key Laboratory of Cancer Prevention and Therapy Tianjin China

**Keywords:** chemoimmunotherapy, immune checkpoint inhibitors, neoadjuvant therapy, non‐small cell lung cancer, surgery

## Abstract

**Background:**

Although neoadjuvant chemoimmunotherapy has emerged as a promising approach for resectable non‐small cell lung cancer (NSCLC), comparative real‐world data on different PD‐1 inhibitors are limited. This study compared the clinical efficacy, pathological response, survival, and safety of four PD‐1 inhibitors—pembrolizumab, tislelizumab, camrelizumab, and sintilimab—in patients with Stage II–IIIa NSCLC.

**Methods:**

We retrospectively reviewed 199 patients with resectable Stage II–IIIa NSCLC treated with neoadjuvant PD‐1 inhibitors plus platinum‐based chemotherapy from January 2018 to December 2024. After excluding 50 non‐surgical cases, 149 patients were included. Outcomes compared included pathological response (pathological complete response, pCR; major pathological response, MPR), recurrence, disease‐free survival (DFS), overall survival (OS), and adverse events.

**Results:**

pCR and MPR rates were 52.2% and 58.0% (pembrolizumab), 67.6% and 75.7% (tislelizumab), 71.4% and 71.4% (camrelizumab), and 47.2% and 61.1% (sintilimab), respectively. Differences in pCR/MPR were not statistically significant. However, OS differed significantly across groups (*p* < 0.05), favoring pembrolizumab and tislelizumab. No significant differences were observed in progression‐free survival (PFS) or recurrence among patients with pCR. Grade ≥ 3 treatment‐related adverse events occurred in 27.0%–42.9% of patients, lowest in the tislelizumab group.

**Conclusion:**

All treatment regimens elicited substantial pathological responses and exhibited acceptable safety profiles. Pembrolizumab and tislelizumab were associated with better OS and lower toxicity, supporting their preferential use in neoadjuvant therapy for resectable NSCLC.

## Graphical abstract

In 149 real‐world patients with resectable Stage II–IIIa NSCLC, neoadjuvant PD‐1 inhibitors (pembrolizumab, tislelizumab, camrelizumab, and sintilimab) plus platinum chemotherapy achieved pCR rates of 47.2%–71.4% with Grade ≥ 3 TRAEs ranging from 27.0%–42.9%. Tislelizumab (HR: 0.167) and sintilimab (HR: 0.090) were associated with superior OS compared with pembrolizumab (reference); camrelizumab showed a nonsignificant trend. Pembrolizumab and tislelizumab demonstrated a favorable balance of efficacy and safety, supporting their preferential use in the neoadjuvant setting.

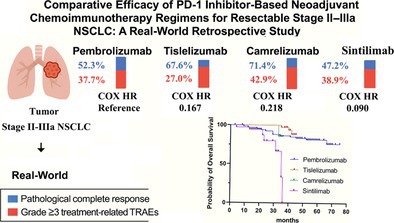

## 1 Introduction

Non‐small cell lung cancer (NSCLC) remains the leading cause of cancer‐related mortality worldwide. Patients with Stage II–III NSCLC represent a significant subset of resectable cases, yet long‐term prognosis remains poor due to high rates of postoperative recurrence and distant metastasis [1]. Although neoadjuvant chemotherapy has historically been employed to improve surgical outcomes, its survival benefit is modest, with an absolute 5‐year overall survival (OS) gain of only approximately 5%, and its utility is limited by toxicity and interpatient variability [2].

In recent years, the emergence of immune checkpoint inhibitors (ICIs), particularly those targeting the programmed cell death protein‐1 (PD‐1) or its ligand PD‐L1, has revolutionized the treatment landscape of NSCLC. Several studies have shown that neoadjuvant immunotherapy combined with chemotherapy significantly improves pathological complete response (pCR), major pathological response (MPR), and long‐term survival endpoints such as disease‐free survival (DFS) and OS [3, 4]. The CheckMate 816 trial notably demonstrated that neoadjuvant nivolumab plus platinum‐doublet chemotherapy led to a pCR rate of 24%, compared with only 2.2% in the chemotherapy‐alone group, with significantly prolonged event‐free survival (EFS) and a trend toward improved OS [3].

Real‐world studies have further validated these findings in broader, less selected populations. For instance, multicenter cohort studies from China have reported pCR rates exceeding 30% and 1‐year PFS as high as 91% among patients receiving neoadjuvant chemoimmunotherapy [5, 6]. These data support the incorporation of neoadjuvant immunotherapy into standard perioperative management for resectable NSCLC.

However, despite the growing adoption of neoadjuvant ICIs, there remains a lack of head‐to‐head studies comparing the efficacy and safety profiles of different PD‐1 inhibitors such as pembrolizumab, tislelizumab, camrelizumab, and sintilimab. Each of these agents is commonly used in China, and differences in their molecular design (e.g., Fc receptor binding), pharmacokinetics, and cost may influence treatment outcomes [7]. Moreover, drug selection in clinical practice is often based on PD‐L1 expression, tumor histology, or clinician preference, rather than direct comparative evidence, posing a challenge to individualized treatment planning.

## 2 Methods

### 2.1 Study Design and Patient Selection

This was a retrospective cohort study based on real‐world clinical data. This retrospective study involving human participants was reviewed and approved by the ethics committee of TJMUCH (approval number: bc2024032) and the institutional review board of TJMUCH (approval number: bc2024032) following the Declaration of Helsinki (as revised in 2013). Informed consent was obtained from all patients before the onset of data analysis. Patients with clinically staged II–IIIc NSCLC who were initially considered resectable by a multidisciplinary thoracic oncology team were consecutively enrolled at Tianjin Medical University Cancer Institute and Hospital between January 1, 2018 and December 31, 2024.

All patients received neoadjuvant immunotherapy combined with platinum‐based chemotherapy (NICT), with regimens selected at the discretion of the treating physicians. No predefined interventions or randomization were applied, thereby reflecting routine clinical decision‐making in thoracic oncology.

All patients had histologically confirmed NSCLC, and clinical staging was determined according to the 8th edition of the TNM classification system by the International Association for the Study of Lung Cancer (IASLC) [8]. From the initial cohort, patients who completed neoadjuvant therapy but did not undergo surgical resection were excluded. Only those who proceeded to curative‐intent surgery with complete resection (R0) were included in the final analysis cohort.

### 2.2 Inclusion and Exclusion Criteria

#### 2.2.1 Initial Inclusion Criteria (for the Total Enrolled Cohort)

Age ≥ 18 years.

Histologically confirmed NSCLC.

Clinical Stage II–IIIc disease at initial diagnosis.

Scheduled to receive neoadjuvant ICI therapy plus platinum‐based chemotherapy.

#### 2.2.2 Exclusion Criteria

Prior history of other active malignancies.

Receipt of radiotherapy or nonstandard neoadjuvant regimens.

Insufficient clinical data or early loss to follow‐up.

#### 2.2.3 Criteria for Final Analytic Cohort ( *n* = 149)

Completion of ≥ 1 cycle of neoadjuvant chemoimmunotherapy.

Underwent surgical resection.

Availability of pathological, survival, and follow‐up data.

### 2.3 Data Collection and Processing

Patient data were extracted from electronic medical records and included the following domains:

Demographic variables: age, sex, smoking status.

Tumor characteristics: histological subtype, PD‐L1 expression status, clinical TNM stage.

Treatment details: PD‐1 inhibitor used, chemotherapy regimen, number of treatment cycles, and immune‐related adverse events.

Surgical data: type of surgery (minimally invasive vs. open thoracotomy), surgical completeness, postoperative complications, length of hospital stay.

Pathologic response: final pathological TNM staging, pathologic complete response (pCR; 0% residual viable tumor [RVT]), and MPR (≤ 10% RVT) [9].

Survival outcomes: disease recurrence, DFS, and OS.

Surgical approach was classified as “minimally invasive” (robot‐assisted or thoracoscopic) or “open thoracotomy.” Pathological response was assessed according to the original pathology reports prepared by board‐certified thoracic pathologists as part of standard clinical practice.

### 2.4 Outcome Measures

#### 2.4.1 Primary Outcomes

pCR rate.

MPR rate.

#### 2.4.2 Secondary Outcomes

R0 resection rate.

Incidence of postoperative complications.

Progression‐free survival (PFS).

OS.

### 2.5 Statistical Analysis

All statistical analyses were performed using SPSS software version 26.0 (IBM Corp.). Categorical variables were reported as counts and percentages, and compared using the chi‐squared test or Fisher's exact test, as appropriate. Continuous variables were expressed as mean ± standard deviation (SD) or median with interquartile range (IQR) and analyzed using the independent *t*‐test or Mann–Whitney *U* test, based on data distribution.

Survival curves were estimated using the Kaplan–Meier method, and differences between groups were evaluated with the log–rank test. Multivariate Cox proportional hazards models were used to identify independent predictors of pCR, DFS, and OS. Survival time calculation: Median follow‐up time was calculated using the reverse Kaplan–Meier method. Multivariable Cox proportional hazards regression models were adjusted for age, sex, histology, clinical stage, and platinum agent to estimate hazard ratios (HRs) with 95% confidence intervals. All statistical tests were two‐sided, and a *p* value < 0.05 was considered statistically significant.

## 3 Results

### 3.1 Patient Cohort and Treatment Assignment

**FIGURE 1:**
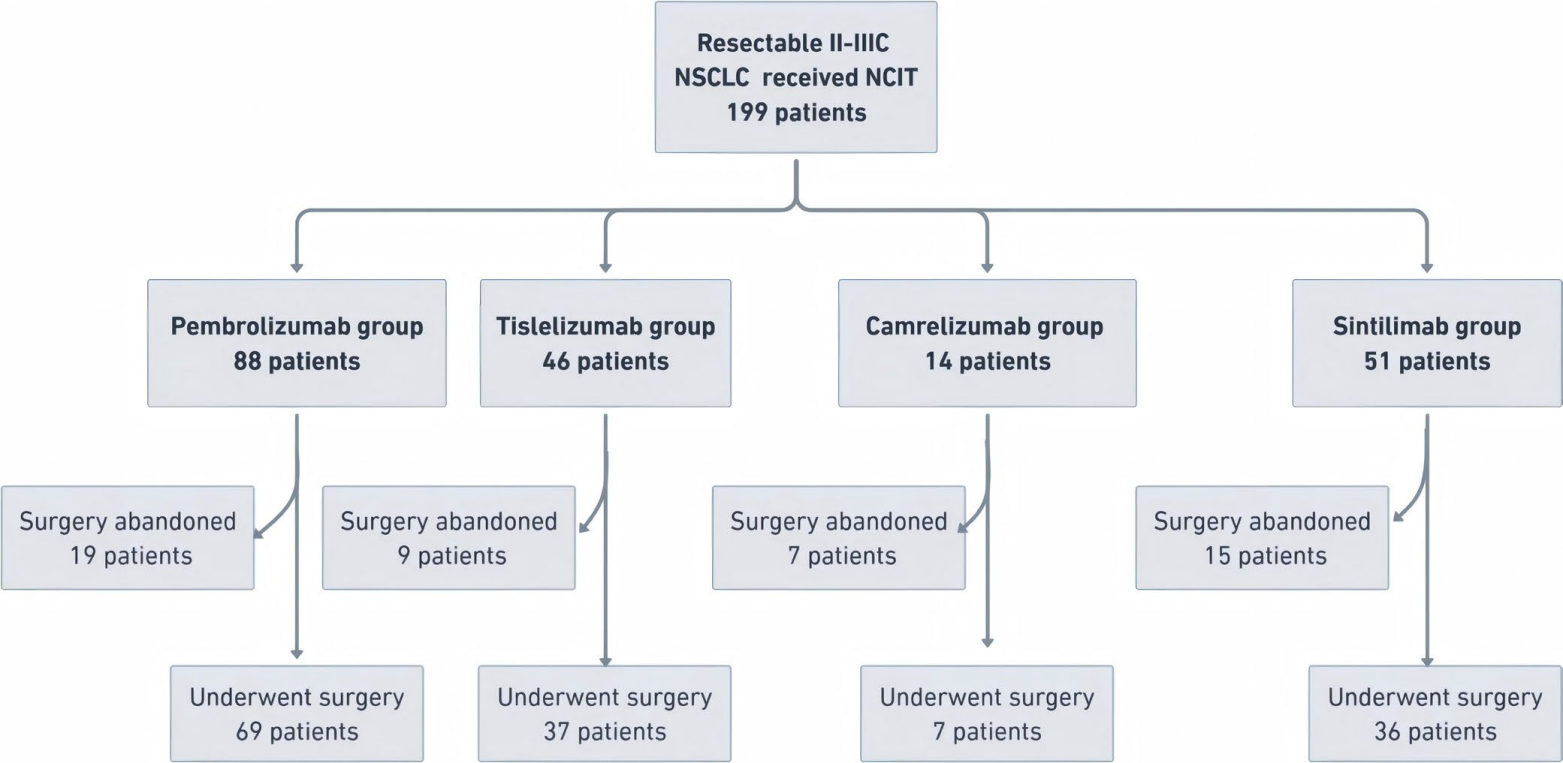
Study flowchart outlining patient enrollment, exclusion, and group assignment. A total of 199 patients with resectable Stage II–IIIa non‐small cell lung cancer (NSCLC) who received neoadjuvant chemoimmunotherapy were screened. After excluding 50 patients who did not proceed to surgery due to disease progression, treatment‐related complications, or patient refusal, 149 patients were included in the final analysis and categorized into four groups according to the PD‐1 inhibitor used.

From January 2018 to December 2024, 199 patients with Stage II–IIIc NSCLC initially assessed as resectable received NICT at Tianjin Medical University Cancer Institute and Hospital. Among them, 50 patients did not proceed to surgery due to patient refusal (*n* = 15), re‐evaluation indicating inoperability (*n* = 12), disease progression (*n* = 19), or mortality (*n* = 4). The final analysis included 149 patients who underwent curative‐intent surgical resection (Figure 1). Patients were stratified by PD‐1 inhibitor used: pembrolizumab (*n* = 69), tislelizumab (*n* = 37), camrelizumab (*n* = 7), and sintilimab (*n* = 36).

In the camrelizumab group, 14 patients were initially enrolled, but only 7 ultimately underwent surgical resection. The other 7 were excluded due to non‐surgical outcomes: 3 patients were deemed inoperable upon re‐evaluation, 2 declined surgery, and 1 died prior to operation. Given the high attrition rate and potential bias, we performed a sensitivity analysis excluding the camrelizumab group to evaluate the robustness of our findings. As shown in Table S1, the exclusion had minimal impact on key clinical outcomes, with comparable rates of pCR, MPR, and estimated 3‐year OS. These results support the stability and reliability of our primary conclusions.

### 3.2 Baseline Characteristics

The baseline clinical and pathological features were largely balanced among the four groups (Table 1). Median age ranged from 60.3 to 62.7 years (*p* = 0.5108), and the majority were male (≥ 86.1%). Squamous cell carcinoma was the predominant histologic type across all groups (*p* = 0.4592). Most patients presented with clinical Stage III disease, and N2 nodal status was observed in over half the patients in each group (range: 57.1%–66.7%, *p* = 0.1166).

**TABLE 1:** Baseline clinical and pathological characteristics of patients across four PD‐1 inhibitor groups.

Characteristics	Pembrolizumab (*n* = 69) *n* (%)	Tislelizumab (*n* = 37) *n* (%)	Camrelizumab (*n* = 7) *n* (%)	Sintilimab (*n* = 36) *n* (%)	*p*
Age					
Mean ± SD	61.21 ± 14.15	62.63 ± 10.45	60.3 ± 4.9	62.68 ± 7.71	0.5108
< 65	39 (62.9)	26 (70.2)	5 (71.43)	19 (52.78)	
≥ 65	23 (37.1)	11 (29.73)	2 (28.57)	17 (47.22)	
Sex					
Female	6 (8.7)	5 (13.51)	0 (0)	5 (13.89)	0.6122
Male	63 (91.3)	32 (86.49)	7 (100)	31 (86.11)	
Pathologic category					
Squamous cell carcinoma	51 (73.9)	31 (83.8)	6 (85.7)	25 (69.4)	0.4592
Adenocarcinoma	14 (20.3)	5 (13.5)	1 (14.3)	8 (22.2)	0.7656
Others	4 (5.8)	1 (2.7)		3 (8.3)	
Clinical T stage					
T1c	5 (7.2)	4 (10.8)	0	2 (5.6)	0.7236
T2a	30 (43.5)	9 (24.3)	1 (14.3)	11 (30.6)	
T2b	10 (14.5)	7 (18.9)	1 (14.3)	6 (16.7)	
T3	16 (23.2)	10 (27.0)	3 (42.9)	9 (25.0)	
T4	8 (11.6)	7 (18.9)	2 (28.57)	8 (22.2)	
Clinical N stage					
N0	7 (10.1)	10 (27.0)	1 (14.3)	10 (27.8)	0.1166
N1	13 (18.8)	4 (10.8)	0	3 (8.3)	
N2	40 (58.0)	22 (59.5)	6 (85.7)	21 (58.3)	
N3	9 (12.0)	1 (2.7)	0	2 (5.6)	
Clinical stage					
IIA	5 (7.2)	7 (18.9)	1 (14.3)	6 (16.7)	0.6037
IIB	11 (15.9)	4 (10.8)	0	3 (8.3)	
IIIA	29 (42.0)	12 (32.4)	2 (28.57)	14 (38.9)	
IIIB	20 (29.0)	10 (27.0)	2 (28.57)	9 (25.0)	
IIIC	4 (5.8)	4 (10.8)	2 (28.57)	4 (11.1)	
Smoking status					
Never	16 (23.2)	10 (27.0)	1 (14.3)	10 (27.8)	0.8543
Former or current	53 (76.8)	27 (73.0)	6 (85.7)	26 (72.2)	
Platinum drugs					
Cisplatin	56 (81.2)	13 (35.1)	0	2 (5.6)	< 0.01
Carboplatin	10 (14.5)	22 (59.5)	6 (85.7)	25 (69.4)	
Others	3 (4.3)	2 (5.4)	1 (14.3)	9 (25.0)	

Notably, platinum agents varied significantly between groups (*p* < 0.01): Cisplatin was favored in the pembrolizumab group, whereas carboplatin was more common in the tislelizumab and sintilimab groups. Camrelizumab was often paired with non‐standard agents (e.g., lobaplatin, nedaplatin).

### 3.3 Surgical and Perioperative Outcomes

All 149 patients proceeded to surgical resection. The R0 resection rate exceeded 83% in all groups and was highest with tislelizumab (94.6%, *p* = 0.2165) (Table 2). Minimally invasive approaches (VATS/RATS) were more frequently used in the tislelizumab (51.4%) and sintilimab (41.7%) groups (*p* = 0.0242), whereas open thoracotomy predominated in the pembrolizumab and camrelizumab groups.

**TABLE 2:** Neoadjuvant treatment and surgical outcomes.

Variables	Pembrolizumab (*n* = 69) *n* (%)	Tislelizumab (*n* = 37) *n* (%)	Camrelizumab (*n* = 7) *n* (%)	Sintilimab (*n* = 36) *n* (%)	*p*
R0 resection (%)					
No	7 (10.1)	2 (5.4)	2 (28.6)	6 (16.7)	0.2165
Yes	62 (89.9)	35 (94.6)	5 (71.4)	30 (83.3)	
Surgical procedure					
VATS/RATS	16 (23.2)	19 (51.4)	2 (28.6)	15 (41.7)	0.0242
Open thoracotomy	53 (76.8)	18 (48.7)	5 (71.4)	21 (58.3)	
Operation time (min, mean ± SD)	93.13 ± 28.59	82.19 ± 22.76	102.57 ± 30.88	97.28 ± 4.18	0.0919
Intraoperative blood loss (ml, mean ± SD)	226.09 ± 147.43	171.62 ± 147.92	235.71 ± 89.97	270.83 ± 226.58	0.0303
Changes of ypTNM stage					
Downstaging	35 (50.8)	21 (56.8)	5 (71.4)	16 (44.4)	0.5212
No change in stage	34 (49.3)	16 (43.2)	2 (28.6)	20 (55.6)	
Postoperative hospital stay (days, mean ± SD)	3.91 ± 1.35	3.43 ± 1.42	4.71 ± 2.14	3.47 ± 1.03	
Postoperative complications^a^ (*n*, %)	6 (8.7)	4 (10.8)	2 (28.6)	4 (11.1)	0.4526
Readmission within 30 days (*n*, %)	3 (4.3)	1 (2.7)	0	1 (2.78)	0.9104
30 days death (%)	0	0	0	0	

Abbreviation: VATS, video‐assisted thoracoscopic surgery.

^a^ Postoperative complications: pneumonia, chylothorax, hydropneumothorax.

There were no significant differences in operative time (*p* = 0.0919). Blood loss was significantly lower in the tislelizumab group (171.6 ± 147.9 mL, *p* = 0.0303). Pathologic downstaging occurred in 50.8%–71.4% of patients, with no intergroup differences (*p* = 0.5212).

Postoperative hospital stays were comparable (mean range: 3.43–4.71 days), with no 30‐day mortality. Complication rates ranged from 8.7% to 28.6% (*p* = 0.4526), and 30‐day readmission occurred in 2.7%–4.3% of patients.

### 3.4 Pathologic Response

pCR was achieved in 36 (52.2%) patients in the pembrolizumab group, 25 (67.6%) in the tislelizumab group, 5 (71.4%) in the camrelizumab group, and 17 (47.2%) in the sintilimab group (*p* = 0.2397). MPR was observed in 58.0%, 75.7%, 71.4%, and 61.1% of patients, respectively (*p* = 0.3129) (Table 3).

**TABLE 3:** Pathological response and recurrence after neoadjuvant chemoimmunotherapy.

Variables	Pembrolizumab (*n* = 69) *n* (%)	Tislelizumab (*n* = 37) *n* (%)	Camrelizumab (*n* = 7) *n* (%)	Sintilimab (*n* = 36) *n* (%)	*p*
pCR status					
No	33 (47.8)	12 (32.4)	2 (28.6)	19 (52.8)	0.2397
Yes	36 (52.2)	25 (67.6)	5 (71.4)	17 (47.2)	
MPR status					
No	29 (42.0)	9 (24.3)	2 (28.6)	14 (38.9)	0.3129
Yes	40 (58.0)	28 (75.7)	5 (71.4)	22 (61.1)	

**FIGURE 2:**
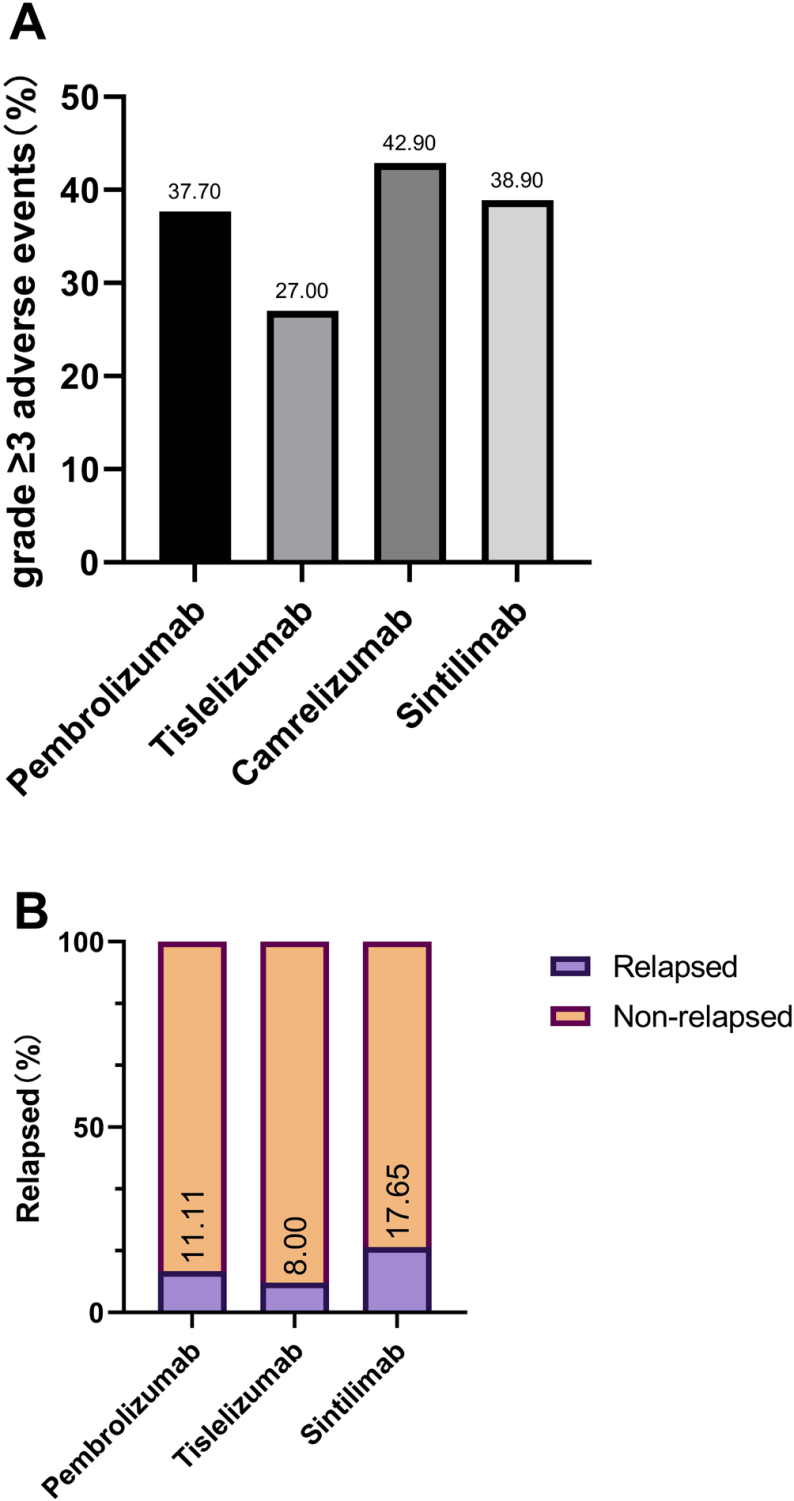
Clinical safety and recurrence outcomes across PD‐1 treatment groups. (A) Recurrence among patients who achieved pathological complete response (pCR) following neoadjuvant chemoimmunotherapy. The bar chart illustrates the number and percentage of pCR patients who developed recurrence in each treatment group: pembrolizumab (4/36, 11.1%), tislelizumab (2/25, 8.0%), camrelizumab (0/5, 0%), and sintilimab (3/17, 17.6%). No statistically significant difference in recurrence rates was observed among the four groups (*p* = 0.5664). (B) Incidence of Grade ≥ 3 treatment‐related adverse events (TRAEs) in each PD‐1 inhibitor group. The bar chart displays the proportion of patients experiencing Grade ≥ 3 TRAEs: pembrolizumab (37.7%), tislelizumab (27.0%), camrelizumab (42.9%), and sintilimab (38.9%). Pairwise comparisons revealed no significant differences in the incidence of severe adverse events between groups.

Among patients who achieved pCR, the incidence of disease recurrence was 11.1% in the pembrolizumab group, 8.0% with tislelizumab, 0% with camrelizumab, and 17.6% with sintilimab (*p* = 0.5664; Figure 2A).

### 3.5 Safety Profile

Grade ≥ 3 treatment‐related adverse events (TRAEs) occurred in 40.0% (pembrolizumab), 27.0% (tislelizumab), 42.9% (camrelizumab), and 38.9% (sintilimab) (*p* = 0.2044 and 0.3257 for intergroup comparisons, Figure 2B). Hematologic toxicities and transaminase elevations were most frequent. All TRAEs were clinically manageable, and no treatment‐related mortality was observed.

### 3.6 Survival Analysis

**FIGURE 3:**
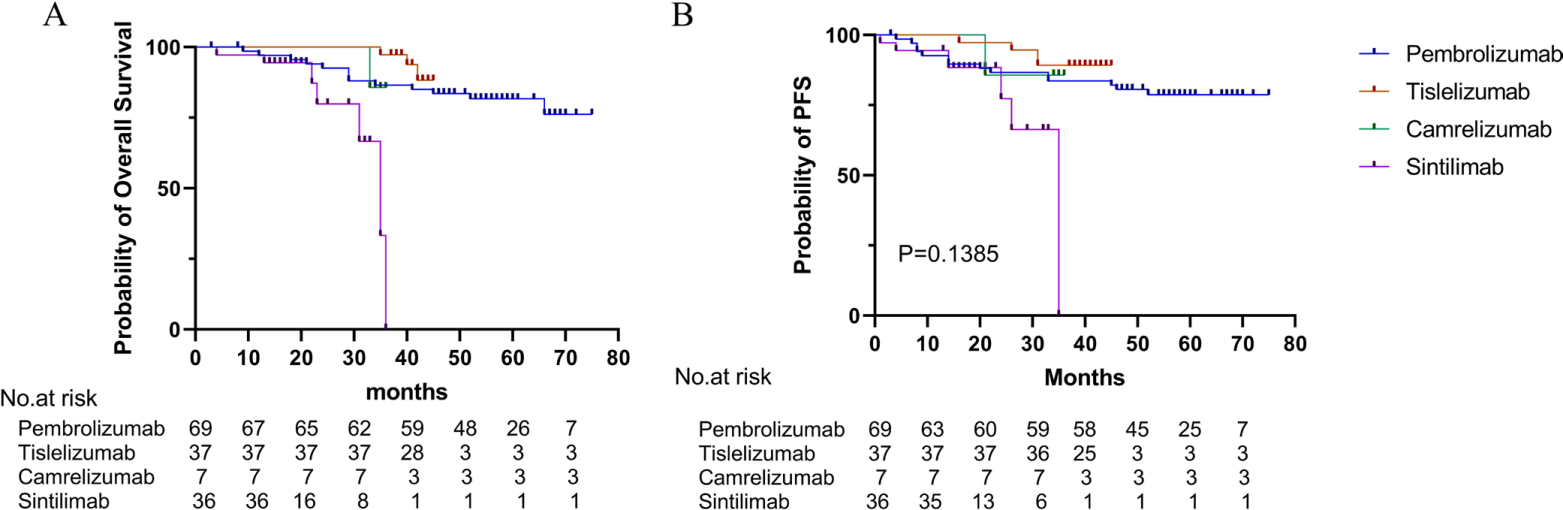
Kaplan–Meier survival curves of progression‐free survival (PFS) and overall survival (OS) across treatment groups. (A) PFS curves show similar trends among groups, with no statistically significant differences (*p* > 0.05). (B) OS curves indicate significantly prolonged survival in the pembrolizumab and tislelizumab groups compared with camrelizumab and sintilimab (*p* < 0.05). Number‐at‐risk tables are presented below each plot.

Kaplan–Meier analysis revealed no significant difference in PFS across treatment arms (*p* > 0.05, Figure 3A). However, numerically superior PFS curves were observed in the pembrolizumab and tislelizumab groups, with prolonged disease control over time. The PFS curves for sintilimab and camrelizumab demonstrated earlier declines compared to other treatment arms.

In contrast, OS differed significantly among groups (*p* < 0.05, Figure 3B). The pembrolizumab and tislelizumab cohorts demonstrated longer OS, with survival plateaus beyond 24 months. The sintilimab group showed an earlier decrease in OS, whereas the camrelizumab group maintained 85.7% survival during the follow‐up period; however, interpretation is limited due to the small sample size (*n* = 7).

The median follow‐up time for the entire cohort was 44 months (IQR: 36.0–58.0 months). Among 149 patients, 125 (83.9%) were censored at last follow‐up, including 120 patients who were alive without recurrence and 5 patients who were lost to follow‐up. The camrelizumab group had a mean follow‐up duration of 35.6 months (95% CI: 34.8–36.3 months) based on the reverse Kaplan–Meier method, with a censoring rate of 6/7 (85.7%). To address concerns regarding survival curve interpretation and sample size limitations, we provided a summary of median OS and HRs derived from multivariable Cox regression (Table S3). Median OS was not reached in any treatment group during the follow‐up period. Compared with the pembrolizumab group (reference), both tislelizumab (HR: 0.167, 95% CI: 0.055–0.508, *p* = 0.002) and sintilimab (HR: 0.090, 95% CI: 0.021–0.384, *p* = 0.001) were associated with significantly improved OS in multivariable analysis. The camrelizumab group (HR: 0.218, 95% CI: 0.026–1.844, *p* = 0.162) showed a favorable trend, but the result was not statistically significant, likely due to small sample size and high censoring rate. These findings reinforce the primary conclusion that PD‐1 inhibitor choice may influence long‐term outcomes, particularly for pembrolizumab and tislelizumab, and highlight the need for further validation in prospective studies.

To assess whether the choice of platinum agent influenced OS, we conducted a stratified survival analysis comparing cisplatin‐ versus carboplatin‐based regimens in all patients. The Kaplan–Meier curves showed no significant difference in OS between the two groups (*p* = 0.88) (Figure S1). These results suggest that the type of platinum agent did not independently affect long‐term survival outcomes in this cohort.

## 4 Discussion

In this real‐world retrospective study, we comprehensively compared the efficacy, pathological response, survival outcomes, and safety profiles of four PD‐1 inhibitors—pembrolizumab, tislelizumab, camrelizumab, and sintilimab—when combined with platinum‐based chemotherapy as neoadjuvant therapy in patients with resectable Stage II–IIIa NSCLC. Although prospective trials such as CheckMate 816 established the benefit of neoadjuvant immunotherapy over chemotherapy alone [3], direct comparative data among different PD‐1 inhibitors in real‐world settings remain extremely limited. Our study provides critical, head‐to‐head, real‐world evidence addressing an urgent clinical question.

Consistent with the CheckMate 816 trial and recent real‐world studies [10, 11], all four regimens achieved substantial pathological responses (pCR range: 47.2%–71.4%, MPR range: 58.0%–75.7%). Notably, although pCR and MPR rates were comparable among groups, pembrolizumab and tislelizumab demonstrated superior long‐term OS. This finding is aligned with recent meta‐analyses demonstrating that neoadjuvant ICI‐based regimens reduce mortality compared with chemotherapy alone [12], and highlights that pathological response may not fully capture survival benefit, especially when drug‐specific immune modulation properties are considered.

Among all agents, tislelizumab showed the most favorable balance of efficacy and safety, likely attributed to its Fc‐engineered IgG4 backbone minimizing Fcγ receptor binding and Treg activation [7]. Structural and mechanistic differences between PD‐1 inhibitors, as suggested by network meta‐analyses [13], could contribute to observed efficacy variation. The relatively lower incidence of Grade ≥ 3 TRAEs with tislelizumab in our cohort may have facilitated better treatment adherence, timely surgery, and thus contributed to a survival advantage.

In contrast, although camrelizumab achieved the highest pCR rate (71.4%), it was associated with a higher rate of immune‐related toxicities impacting surgical eligibility. Among the first 14 patients treated with camrelizumab at our center, 7 were unable to proceed to surgery, predominantly due to hepatotoxicity and RCCEP‐induced treatment modification [14]. These findings underscore the practical importance of tolerability, not just pathological efficacy, when selecting neoadjuvant immunotherapy regimens.

The sintilimab group showed relatively less favorable OS outcomes. Although early‐phase trials, such as ORIENT‐11, support sintilimab's efficacy in advanced NSCLC [15], its role in the neoadjuvant setting remains less well defined. Factors such as different chemotherapy backbones, patient selection, pharmacokinetics, or immune activation profiles may contribute to these differences. Further dedicated studies are needed.

Although pathological response has often been used as a surrogate endpoint for survival, our findings indicate that the relationship between pCR and OS is influenced by multiple factors. Trials such as CheckMate 816 and NADIM have shown improved EFS and OS despite modest pCR rates (24% and 63.4%, respectively) [3, 16], whereas KEYNOTE‐671 demonstrated an 18% pCR rate but achieved significant OS benefit [17]. Conversely, our study found relatively high pCR rates but OS benefit only in certain PD‐1 subgroups. These discrepancies may be partly explained by differences in study population, stage distribution, adjuvant therapy use, and drug‐specific mechanisms. For example, NADIM enrolled only Stage IIIA patients and mandated adjuvant nivolumab, whereas our real‐world cohort had broader staging and variable adjuvant strategies. Moreover, emerging biomarkers such as ctDNA clearance after neoadjuvant therapy have shown strong correlation with OS in trials such as NADIM [16], suggesting their potential utility beyond pCR. Therefore, although pCR is valuable, its prognostic utility may vary depending on patient characteristics, treatment strategy, and follow‐up duration.

A notable strength of our study is that, unlike most RCTs, we did not exclude patients with actionable driver mutations (e.g., EGFR, ALK). This decision reflects real‐world practice where patients with oncogenic mutations—especially those with high PD‐L1 or urgent clinical needs—often still receive chemoimmunotherapy. Recent retrospective analyses [4, 18] suggest that some EGFR‐mutant NSCLC patients, particularly those with high PD‐L1 expression, may still benefit, although results remain heterogeneous.

Pathological response proved to be a strong surrogate for survival in our study, echoing findings from NADIM, NADIM II [16], CheckMate 816 [3], and KEYNOTE‐671 [17], which all confirmed that achieving MPR or pCR correlates with improved DFS and OS. Furthermore, Hellmann et al. [9] highlighted that each 10% increase in RVT raises recurrence risk by 17%. These reinforce the role of pathological response, but our data also suggest that long‐term survival benefit depends on both response and treatment tolerability.

From a safety perspective, the incidence of Grade ≥ 3 TRAEs (27.0%–42.9%) was comparable to those reported in CheckMate 816 and other phase II trials [3, 16]. Importantly, the relatively lower toxicity of tislelizumab may have contributed to superior OS by enabling better completion of neoadjuvant therapy and timely surgery. These findings highlight that balancing efficacy and tolerability is crucial when optimizing perioperative strategies.

Although recent real‐world studies have evaluated neoadjuvant chemoimmunotherapy, including Fang et al. [11] and Hu et al. [10], our study contributes novel insights. Fang et al. reported pCR 37.9% in a mixed‐cohort study involving six PD‐1 inhibitors without direct comparison. Hu et al. compared pembrolizumab and tislelizumab but had a smaller sample (*n* = 126) and limited observation time. In contrast, we included four PD‐1 agents, analyzed complete surgical and survival outcomes, evaluated irAEs' impact on surgery, and incorporated patients with molecular heterogeneity, thus better reflecting the complexity of real‐world clinical practice.

Although PD‐L1 expression and driver mutations (e.g., EGFR, ALK) are established predictive biomarkers for ICIs in advanced NSCLC, these factors were not systematically analyzed in the present study due to data incompleteness. Notably, our cohort was predominantly squamous cell carcinoma, in which EGFR/ALK mutations are rare, minimizing their likely impact. PD‐L1 expression was also unavailable in a substantial number of cases, limiting stratified analysis. Importantly, our prior real‐world studies involving similar treatment settings found no significant association between PD‐L1 levels and either pathological response or survival outcomes [19]. These findings are consistent with recent clinical trials of neoadjuvant chemoimmunotherapy—such as CTONG1804 (NCT04015778)—which demonstrated that the pathological response (MPR/pCR) and survival benefit (EFS) of combined chemotherapy were not related to the expression level of PD‐L1. Thus, in the neoadjuvant setting, PD‐L1 may have limited predictive value. Future prospective trials incorporating comprehensive molecular profiling (e.g., PD‐L1, EGFR, ALK, ctDNA) are warranted to refine patient selection and optimize therapeutic strategies.

Finally, our findings provoke important biological questions. For instance, although CheckMate 816 reported relatively low pCR rates (24%) but positive EFS benefit [3], our study showed that pembrolizumab achieved both high pCR and favorable OS. This discrepancy may reflect differences in cohort characteristics, surgical management, timing of resection, or true biological variability in drug–tumor interactions. Future biomarker‐driven studies are warranted to refine neoadjuvant strategies.

## 5 Conclusion

This real‐world retrospective study provides comparative evidence for the use of four PD‐1 inhibitors combined with platinum‐based chemotherapy in the neoadjuvant setting for resectable Stage II–IIIa NSCLC. All regimens demonstrated substantial pathological responses and manageable safety profiles. Pembrolizumab and tislelizumab were associated with superior long‐term survival and lower incidence of severe adverse events. These findings emphasize that individualized treatment selection, considering both efficacy and tolerability, is critical in optimizing outcomes for NSCLC patients. Future prospective, biomarker‐guided trials are needed to further refine perioperative immunotherapy strategies.

## Author Contributions

Conception and design and administrative support: Yulong Chen. Provision of study material or patients' information: Xiaoxuan Sun. Collection and assembly of data: Yulong Chen, Bo Yan, and Ran Zhang. Data analysis and interpretation: Yulong Chen and Yan Sheng. Manuscript writing: All authors. Final approval of manuscript: All authors. Accountable for all aspects of the work: All authors.

## Conflicts of Interest

The authors declare no conflicts of interest.

## Supporting information


**Figure S1.** Overall survival stratified by platinum agent (cisplatin vs. carboplatin). Kaplan–Meier curves comparing overall survival (OS) in patients treated with cisplatin‐ versus carboplatin‐based regimens. No statistically significant difference in OS was observed between the two groups (*p* = 0.88).

**Table S1.** Sensitivity analysis. This table compares the pathological complete response (pCR), major pathological response (MPR), and 3‐year overall survival (OS) rates between the full analytic cohort (*n* = 149) and a sensitivity cohort excluding the camrelizumab group (*n* = 142). The results indicate that excluding the camrelizumab subgroup did not significantly alter key clinical outcomes, confirming the robustness of the primary analysis.
**Table S2.** pCR rate comparison by platinum agent across PD‐1 inhibitor groups. Comparison of pCR rates by platinum agent type across PD‐1 inhibitor groups. This table presents the number and proportion of patients achieving pCR stratified by cisplatin‐ versus carboplatin‐based regimens among all patients, as well as within the pembrolizumab and sintilimab groups. No significant difference in pCR or OS was observed between the two platinum agents, suggesting that platinum selection did not independently impact treatment efficacy in this cohort.
**Table S3.** Median overall survival (OS) and hazard ratios from multivariable Cox regression model across PD‐1 inhibitor groups. This table summarizes the multivariate survival analysis for each PD‐1 inhibitor group using Cox proportional hazards modeling. Pembrolizumab was used as the reference group. Median OS was not reached in any group during the follow‐up period. Both tislelizumab and sintilimab were associated with significantly lower hazards of death than pembrolizumab, whereas the camrelizumab group showed a favorable trend that did not reach statistical significance, likely due to small sample size and high censoring rate.

## Data Availability

The data that support the findings of this study are available from the corresponding author upon reasonable request.
