# Supplementary figures and images for: Comparative Efficacy of PD‐1 Inhibitor‐Based Neoadjuvant Chemoimmunotherapy Regimens for Resectable Stage II–IIIa NSCLC: A Real‐World Retrospective Study
Source: Thorac Cancer. 2025 Jul 7;16(13):e70123. doi: 10.1111/1759-7714.70123 (PMC12234159; doi:10.1111/1759-7714.70123)

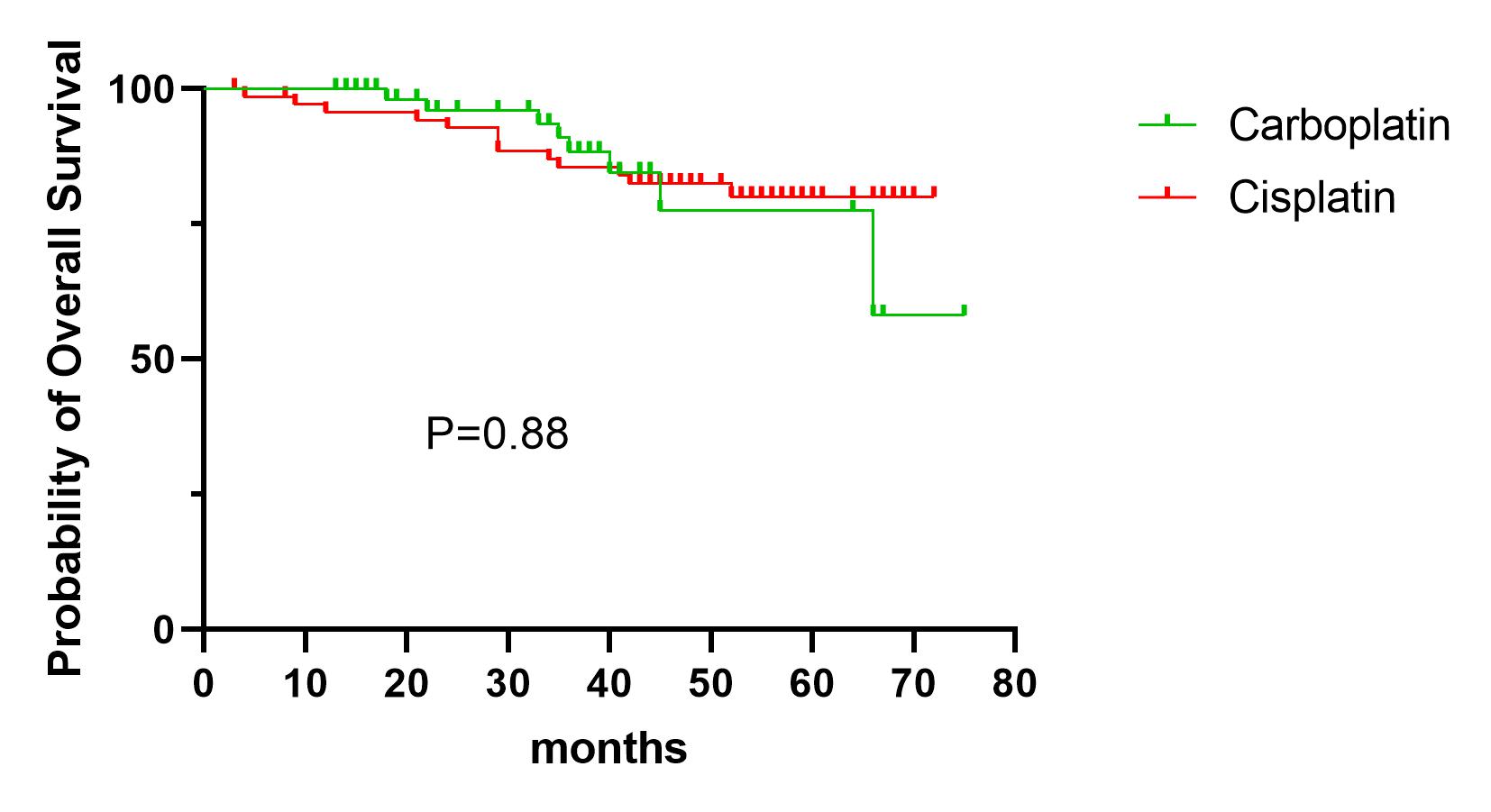

Supplement: Supplementary file 1 — Figure S1. Overall survival stratified by platinum agent (cisplatin vs. carboplatin). Kaplan–Meier curves comparing overall survival (OS) in patients treated with cisplatin‐ versus carboplatin‐based regimens. No statistically significant difference in OS was observed between the two groups (p = 0.88). [file TCA-16-e70123-s002.jpg]
