# Supplementary material for: Comparative Efficacy of PD‐1 Inhibitor‐Based Neoadjuvant Chemoimmunotherapy Regimens for Resectable Stage II–IIIa NSCLC: A Real‐World Retrospective Study
Source: Thorac Cancer. 2025 Jul 7;16(13):e70123. doi: 10.1111/1759-7714.70123 (PMC12234159; doi:10.1111/1759-7714.70123)
Supplement: Supplementary file 2 — Table S1. Sensitivity analysis. This table compares the pathological complete response (pCR), major pathological response (MPR), and 3‐year overall survival (OS) rates between the full analytic cohort (n = 149) and a sensitivity cohort excluding the camrelizumab group (n = 142). The results indicate that excluding the camrelizumab subgroup did not significantly alter key clinical outcomes, confirming the robustness of the primary analysis. Table S2. pCR rate comparison by platinum agent across PD‐1 inhibitor groups. Comparison of pCR rates by platinum agent type across PD‐1 inhibitor groups. This table presents the number and proportion of patients achieving pCR stratified by cisplatin‐ versus carboplatin‐based regimens among all patients, as well as within the pembrolizumab and sintilimab groups. No significant difference in pCR or OS was observed between the two platinum agents, suggesting that platinum selection did not independently impact treatment efficacy in this cohort. Table S3. Median overall survival (OS) and hazard ratios from multivariable Cox regression model across PD‐1 inhibitor groups. This table summarizes the multivariate survival analysis for each PD‐1 inhibitor group using Cox proportional hazards modeling. Pembrolizumab was used as the reference group. Median OS was not reached in any group during the follow‐up period. Both tislelizumab and sintilimab were associated with significantly lower hazards of death than pembrolizumab, whereas the camrelizumab group showed a favorable trend that did not reach statistical significance, likely due to small sample size and high censoring rate. [file TCA-16-e70123-s001.docx]

**Supplementary table 1** Sensitivity Analysis

| Groups | pCR（%） | MPR（%） | 3-Years OS（%） | p Value（pCR） | p Value（OS） |
| --- | --- | --- | --- | --- | --- |
| All patients (n=149) | 83 | 90 | 65.8 | 0.988 | 0.9972 |
| Excluding camrelizumab (n=142) | 78 | 95 | 66.9 |  |  |

**Supplementary table 2** pCR Rate Comparison by Platinum Agent Across PD-1 Inhibitor Groups**.**

| Groups | Cisplatin (n, pCR%) | Carboplatin (n, pCR%) | p Value（OS） |
| --- | --- | --- | --- |
| All patients | 71，56.3 | 63，55.5 | 1.000 |
| Pembrolizumab | 10,40.0 | 56,55.4 | 0.496 |
| Sintilimab | 25，52.0 | 2，0 | 0.481 |

**Supplementary table 3 Median Overall Survival and Hazard Ratios from Multivariable Cox Regression**

| Group | No. of Patients | Median OS (months) | HR vs Pembrolizumab | 95% CI | P value |
| --- | --- | --- | --- | --- | --- |
| Pembrolizumab | 69 | Not reached | Reference | - | - |
| Tislelizumab | 37 | Not reached | 0.167 | 0.055–0.508 | 0.002 |
| Sintilimab | 36 | Not reached | 0.090 | 0.021–0.384 | 0.001 |
| Camrelizumab | 7 | Not reached | 0.218 | 0.026–1.844 | 0.162 |
